# Down-regulation of BTG1 by miR-454-3p enhances cellular radiosensitivity in renal carcinoma cells

**DOI:** 10.1186/1748-717X-9-179

**Published:** 2014-08-12

**Authors:** Xin Wu, Nan Ding, Wentao Hu, Jinpeng He, Shuai Xu, Hailong Pei, Junrui Hua, Guangming Zhou, Jufang Wang

**Affiliations:** Department of Space Radiobiology, Key Laboratory of Heavy Ion Radiation Biology and Medicine, Institute of Modern Physics, Chinese Academy of Sciences, Lanzhou, 509 Nanchang Road, Lanzhou, 730000 China; University of Chinese Academy of Sciences, Beijing, 100049 P.R China

**Keywords:** microRNA, BTG1, miR-454-3p, Radiosensitivity, S phase arrest

## Abstract

**Background:**

*B cell translocation gene 1* (*BTG1*) has long been recognized as a tumor suppressor gene. Recent reports demonstrated that BTG1 plays an important role in progression of cell cycle and is involved in cellular response to stressors. However, the microRNAs mediated regulatory mechanism of BTG1 expression has not been reported so far. MicroRNAs can effectively influence tumor radiosensitivity by preventing cell cycle progression, resulting in enhancement of the cytotoxicity of radiotherapy efficacy. This study aimed to demonstrating the effects of microRNAs on the BTG1 expression and cellular radiosensitivity.

**Methods:**

The human renal carcinoma 786-O cells were treated with 5 Gy of X-rays. Expressions of BTG1 gene and miR-454-3p, which was predicted to target BTG1 by software algorithm, were analyzed by quantitative polymerase chain reaction. Protein expressions were assessed by Western blot. Luciferase assays were used to quantify the interaction between BTG1 3′-untranslated region (3′-UTR) and miR-454-3p. The radiosensitivity was quantified by the assay of cell viability, colony formation and caspase-3 activity.

**Results:**

The expression of the BTG1 gene in 786-O cells was significantly elevated after treatments with X-ray irradiation, DMSO, or serum starvation. The up-regulation of BTG1 after irradiation reduced cellular radiosensitivity as demonstrated by the enhanced cell viability and colony formation, as well as the repressed caspase-3 activity. In comparison, knock down of *BTG1* by siRNA led to significantly enhanced cellular radiosensitivity. It was found that miR-454-3p can regulate the expression of *BTG1* through a direct interaction with the 3′-UTR of *BTG1* mRNA. Decreasing of its expression level correlates well with BTG1 up-regulation during X-ray irradiation. Particularly, we observed that over-expression of miR-454-3p by transfection inhibited the *BTG1* expression and enhanced the radiosensitivity. In addition, cell cycle analysis showed that over-expression of miR-454-3p shifted the cell cycle arrest from G2/M phase to S phase.

**Conclusions:**

Our results indicate that *BTG1* is a direct target of miR-454-3p. Down-regulation of *BTG1* by miR-454-3p renders tumor cells sensitive to radiation. These results may shed light on the potential application in tumor radiotherapy.

**Electronic supplementary material:**

The online version of this article (doi:10.1186/1748-717X-9-179) contains supplementary material, which is available to authorized users.

## Background

Enhancing the radiosensitivity of resistant tumor cells is a common emphasis in the application of clinical radiotherapy [1]. Tumor suppressor genes that regulate cell cycle can alter the radiosensitivity of cancer cells. For example, down-regulation of RB1 correlates with increased apoptosis induced by radiation in human breast cancer cells [2, 3]; the mutations in the p53 tumor suppressor gene enhances the radiosensitivity in oral cavity carcinoma cells [4]; and *P21* deficiency is associated with a radiosensitive phenotype in colorectal carcinoma cells [5]. The *RB1*, *p53* and *p21* are tumor suppressor genes and play cruical roles in controlling the progression of cell cycle [6, 7]. These findings indicate that cell cycle regulation genes may be intimately related to radiosensitization, therefore, could potentially be exploited in tumor radiotherapy. In this study, the human renal carcinoma cell, which has traditionally been considered to be radioresistant [8], was used as experimental model.

*B cell translocation gene 1* (*BTG1*) is another tumor suppressor gene [9, 10]. The *BTG1* protein, together with five additional proteins (BTG2/PC3/Tis21, BTG3/ANA, BTG4/PC3B, Tob1/Tob, and Tob2), comprise the *BTG/TOB* family of anti-proliferative genes involved in the regulation of cell growth [11]. Expression of *BTG1* not only inhibits the proliferation of cells but also leads to G1 phase cell cycle arrest in multiple types of cells [12–14]. Some studies have shown that BTG1 is involved in the general processes of cell cycle control and in cellular responses to stress [15], though a specific role for BTG1 in renal cell carcinoma has not been determined. In consideration of the common physiological function of tumor suppressor genes in controlling cell cycle, we propose that *BTG1* may have a similar impact as *RB1*, *p53*, and *p21* on the radiosensitivity of renal carcinoma tumor cells.

Certain members of the *BTG/TOB* family are known to be regulated by microRNAs (miRNAs) [16], which are small non-coding RNA molecules that suppress gene expression via sequence-specific interactions with the 3′-untranslated region (3′-UTR) of their target transcripts [17]. For example, *BTG2* was shown to be suppressed by miR-21 [18]; over-expression of miR-142-5p leads to down-regulation of *BTG3* [19]; and *TOB2* was shown to be a target gene of miR-322 [20]. However, miRNA candidates that target *BTG1* have not been identified.

The strategy of using miRNAs as therapeutic targets to enhance cellular radiosensitivity has been discussed before [21]. miRNAs can efficiently modulate tumor radiosensitivity at four aspects containing DNA damage repair, radio-related signal transduction pathways, tumor microenvironment and apoptosis [22, 23]. Recent reports show that miRNAs can effectively influence tumor radiosensitivity by impeding cell cycle progression, resulting in enhancement of radiotherapy efficacy [24]. For example, miR-21 can improve tumor radiosensitivity and promote apoptosis through negatively regulating the *CDC25A* expression and cell cycle progression [25]. Up-regulation of miR-504 can reduce *p53* protein level and affect cell cycle arrest and radiosensitivity mediated by p53 [26]. With these precedents, we tested whether the *BTG1* could be regulated by miRNAs upon irradiation and how the cellular radiosensitivity in renal carcinoma cells could be affected by the changes of miRNAs targeting *BTG1*.

## Methods

### Cell culture and irradiation

Human renal carcinoma 786-O cells were cultured in RPMI-1640 media (GIBCO, NY, USA) supplemented with 10% fetal bovine serum (Hyclone, MA, USA) at 37°C and 5% CO_2_. Irradiation was carried out by laboratory X-ray source (RX-650, Faxitron Bioptics, USA) [27]. The dose rate was 0.8 Gy/min (100 keV, 5 mA). Cells were seeded into 12-well plates and grown to <70% confluence at the time of irradiation.

### Cell cycle assay

Cells were harvested and fixed as described previously [28]. Prior to analysis, fixed cells were washed twice with PBS, treated with 100 μg/mL RNase A and 50 μg/mL propidium iodide (BD Biosciences, California, USA) for 20 min, and analyzed using FACS Calibur flow cytometry (Becton Dickinson, NJ, USA). Cell cycle distribution was analyzed with the FlowJo software package. DNA content of samples was measured with CellQuest (Becton Dickinson).

### Western blot analysis

Western blotting was performed as described previously [29]. Briefly, cells were collected and lysed with RIPA buffer solution (Beyotime, Haimen, China). Supernatants were collected at 10,000 g for 10 min at 4°C. The concentrations of samples were determined using a BCA protein assay kit (Pierce, IL, USA). Equal amounts of protein were loaded onto 10% SDS-polyacrylamide gels for electrophoresis, and proteins were transferred onto PVDF membrane by western blotting. GAPDH was used as loading control. Membranes were then blocked with nonfat milk and incubated overnight at 4°C with the following primary antibodies: anti-GAPDH (1:5,000, Santa Cruz), anti-BTG1 (1:1,000, Abnova, Taipei, Taiwan). Immunological complexes were detected by enhanced electrochemiluminescence (Millipore, Darmstadt, Germany) with either an anti-rabbit peroxidase (1:5000, Santa Cruz, Texas, USA), or an anti-mouse peroxidase (1:4000, Santa Cruz) antibody. The fold changes of protein levels were analyzed by the Image J software.

### Transfection

MicroRNA mimic and negative control mimic were synthesized and purified by RiboBio Co. (Guangzhou, China) [29]. Transfections of the miRNA duplexes were performed with 40–60% confluent cells using Lipofectamine 2000 (Invitrogen, California, USA). The medium was replaced with new culture medium for 5 h after transfection.

### Trypan blue dye exclusion assay

Cell viability is calculated as the number of viable cells divided by the total number of cells within the grids on the hemacytometer. Cells which take in trypan blue are considered non-viable. Cells were harvested with trypsin, suspended in PBS and mixed with 0.4% solution of trypan blue stain (Invitrogen) after various treatments. Count at least 500 cells for calculation. The percentage of viable cells = [1.00 - (Number of blue cells/Number of total cells)]*100%.

### Caspase-3 activity assay

To evaluate the activity of caspase-3, the caspase-3 activity kit (Beyotime) was used. Cells were collected and lysed with reaction buffer and the total protein concentration is 1-3 mg/mL. In the samples, activated caspase-3 cleaves substrate (Ac-DEVD-pNA) (2 mM) between DEVD and pNA, quantitatively generating pNA that can be detected using an ELISA reader at an absorbance of 405 nm. In the caspase-3 colorimetric calibration, the value of R^2^ should be greater than 0.999.

### Colony formation assay

The clonogenic assay was conducted as described previously [30]. Briefly, cells were harvested and an appropriated number of cells were seeded onto each of the 60 mm dishes to produce about 50–120 colonies. After 8–10 days incubation, the colonies were washed with 1 × PBS softly, fixed with 70% ethanol for 5 min and stained with 0.5% crystal violet for 3 min at room temperature. Colonies with more than 50 cells were counted.

### Luciferase reporter assay

The 3′-untranslated region (3′-UTR) of human *BTG1* transcript was cloned downstream of the luciferase gene between the Xho I and Sal I sites of the pmirGLO dual-luciferase vector (Promega, WI, USA). A pmirGLO dual-luciferase vector containing one mutated seed sequences of miR-454-3p was constructed. The sequencing of constructed plasmids was verified by Shanghai Sangon Biotechnology Co. (Shanghai, China). 1.5 × 10^5^ 786-O cells in 12-well plate were co-transfected with 300 ng DNA (pmirGLO-3′ UTR constructs or derived mutants) and 30 nM of either miR-454-3p mimics using transfection reagent Lipofectamine 2000 (Invitrogen). Luciferase activity was measured 48 h later using the Dual Luciferase Reporter Assay System (Promega) [31] with a Tecan Infinite M200 Pro microplate reader (Tecan, Mannedorf, Switzerland).

### Quantitative real-time reverse transcription-PCR

For qRT-PCR, total RNAs were extracted from cultured cells using TRIzol Reagent (Invitrogen) according to the manufacturer’s protocol. Reverse transcription and quantitative RT-PCR were performed according to the protocol of the qRT-PCR Detection Kit (Promega). All of the stem-loop RT primers were purchased from RiboBio Co. (Guangzhou, China) to detect miR-454-3p or U6. U6 was used as an endogenous control for miRNAs and GAPDH for coding genes. Other gene-specific primers were as follows: *BTG1*, 5′-TCCATAATCCATCCCCAAGA-3′ and 5′-GGATGCAATCCTGGACATTT-3′, *SKA2*, 5′-CCGCTTTAAACCAGTTGCTG-3′ and 5′-CTCTGCCGCAGTTTTCTCTT-3′, *GAPDH*, 5′-GTGGACCTGACCTGCCGTCT-3′ and 5′-GGAGGAGTGGGTGTCGCTGT-3′. Gene-specific primers were synthesized from Shanghai Sangon Biotechnology Co. (Shanghai, China).

### Statistical analyses

All experiments were repeated at least three times, and data were presented as means ± SE. The statistical significance of the results was determined by Student’s t-tests using Microsoft Excel (Microsoft Campus, Redmond, WA, USA).

## Results

### BTG1 is induced in response to ionizing radiation

**Figure 1:**
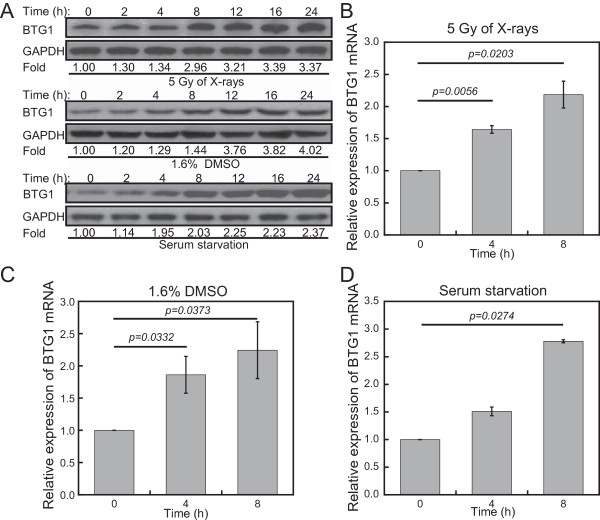
**BTG1 is induced in response to various extracellular stressors in 786-O cells. (A)** Western blot assay of BTG1 at indicated time points after treatment of human renal carcinoma 786-O cells with 5 Gy of X-rays, DMSO (1.6%, v/v), and serum deprivation. GAPDH is used as a loading control. Fold changes of protein levels represent the average of three separate blots. **(B-D)** *BTG1* mRNA levels were monitored by qRT-PCR at 0, 4, and 8 h after treatment of 786-O cells with **(B)** 5 Gy of X-rays, **(C)** DMSO (1.6%, v/v) and **(D)** serum starvation. Values were normalized to GAPDH. Results represent means ± SE of four independent experiments.

Many tumor suppressor genes are recognized as responders to ionizing radiation (IR) [32]. To investigate whether BTG1 functions as a responder to IR, we examined the protein levels of *BTG1* in response to 5 Gy of X-rays in renal carcinoma 786-O cells by Western blot analysis. The protein levels of *BTG1* were significantly increased 8 h after irradiation (Figure 1A). We also treated 786-O cells with DMSO and serum starvation to investigate whether *BTG1* responds to other types of extracellular stressors and a similar increase in BTG1 levels was observed. Consistent with the results of Western blotting, qRT-PCR showed that the mRNA levels of *BTG1* were increased by more than 2-fold upon treatment with irradiation (Figure 1B), DMSO (Figure 1C) or serum starvation (Figure 1D). These results indicate that, like other tumor suppressor genes, *BTG1* is up-regulated in response to X-ray. Meanwhile, the BTG1 can also be induced by other sources of extracelluar stressors, including the treatments by DMSO and serum starvation.

### Tumor cell sensitivity to IR correlates with BTG1 levels

**Figure 2:**
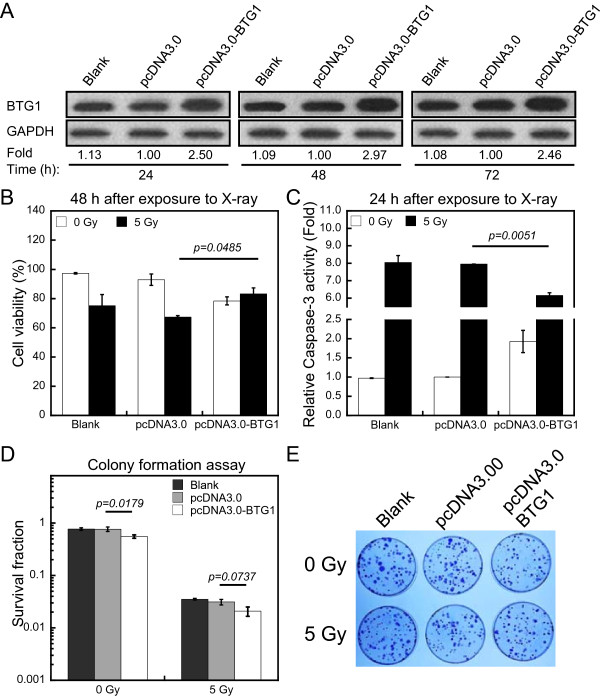
**Tumor cell sensitivity to IR correlates with BTG1 levels. (A)** Western blot showing the up-regulation of BTG1 protein in 786-O cells after transfection with the BTG1 expression vector pcDNA3.0-BTG1 as compared to the control pcDNA3.0 vector and untransfected (blank) cells after 24, 48 and 72 h. GAPDH is used as loading control. Fold changes of protein levels represent the average of three separate blots. **(B)** The trypan blue exclusion assay was used to assess cell viability in 7860-O cells without transfection or after transfection with pcDNA3.0 vector or pcDNA3.0-BTG1 vector at 48 h after X-ray exposure. **(C)** The changes of caspase-3 activity of the three group of 786-O cells was analyzed by caspase-3 activity assay kit at 24 h after X-rays. **(D)** Survival fractions of cells transfected with the control pcDNA3.0 vector and pcDNA3.0-BTG1 vector in response to 0, 5 Gy of X-rays were measured by colony formation assay. **(E)** Photographs of colonies formed by 786-O cells after various treatments. The data (means ± SE) are representative for at least five independent experiments.

Because *BTG1* is involved in the regulation of cell cycle progression and responds to IR, we examined the effects of *BTG1* expression on the radiosensitivity of renal carcinoma 786-O cells. We transfected 786-O cells with the *BTG1* expression vector pcDNA3.0-BTG1 in order to up-regulate the protein levels of *BTG1*. The efficiency of transfection was confirmed by Western blotting (Figure 2A). The protein levels of *BTG1* increased notably at 24 h after transfection and lasted for at least three days.

To estimate radiosensitivity, the cell viability was assessed by trypan blue dye exclusion assay [33]. In this study, transfection of 786-O cells with pcDNA3.0-BTG1 significantly increased cell viability after radiation exposure (Figure 2B). To further confirm the effects of BTG1 on radiosensitivity, we measured the activity of caspase-3 (see Methods) which is an acknowledged effector among the caspase family members involved in apoptosis [34]. In order to more accurately characterize the changes of caspase-3 activity among all treatments, we drew standard curve and tested the caspase-3 activity at 24 h after irradiation (Additional file 1A and B). At 24 h after exposure to 5 Gy of X-rays, compared to transfection of pcDNA3.0, treatment of 786-O cells with transfection of pcDNA3.0-BTG1 caused a significant decrease in caspase-3 activation (Figure 2C). A colony formation assay was performed to determine the cellular radiation sensitivity. The survival fraction of 786-O cells transfected with pcDNA3.0-BTG1 without irradiation was significantly lower than that transfected with pcDNA3.0, due to the fact that *BTG1* is an anti-proliferative gene. It is worth noting that there was no statistically significant difference in the survival fraction of cells exposed to 5 Gy of X-rays, when the group transfected pcDNA3.0-BTG1 was compared with the pcDNA3.0 group (Figure 2D and E). We also used flow cytometry to quantify changes in the sub-G1 peak at 48 h. The peak of transfection of *BTG1* expression vector was decreased upon exposure to IR compared to the untransfected (Blank) group and the pcDNA3.0 group (Additional file 1C). These results suggest that *BTG1* expression protects cells from the cytotoxic effects of IR.

**Figure 3:**
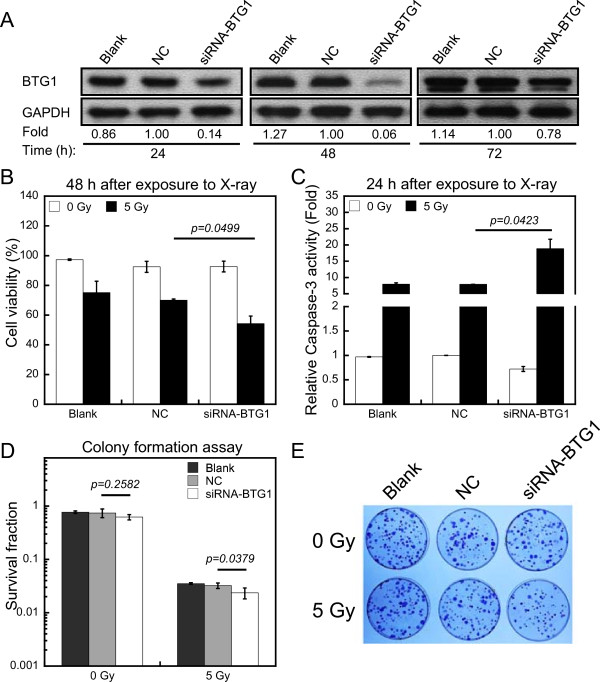
**Down-regulation of** ***BTG1*** **significantly increases cellular radiation sensitivity in 786-O cells. (A)** The down-regulation of endogenous BTG1 protein following transfection with siRNA oligonucleotides against BTG1 (siRNA-BTG1) or nonsense small RNA oligonucleotides (negative control, NC) were compared to untransfected (blank) cells. GAPDH serves as loading control. Fold changes of protein levels represent the average of three separate blots. **(B)** The percentage of 786-O cell viability after transfection with siRNA oligonucleotides against BTG1 (siRNA-BTG1) or nonsense small RNA oligonucleotides (negative control, NC) was determined by trypan blue dye exclusion assay at 48 h after X-ray exposure. **(C)** Effects of nonsense small RNA oligonucleotides or siRNA-BTG1 over-expression on caspase-3 activity in 786-O cells without irradiation or with 5 Gy of X-rays. **(D)** Colony formation assay of 786-O cells transfected with NC or siRNA-BTG1. **(E)** Representative plates of survival fraction of 786-O cells after various treatments. The data (means ± SE) are representative for at least five independent experiments.

To further examine the role of BTG1 in radiosensitivity of 786-O cells, we used siRNA oligonucleotides targeting *BTG1* to specifically knock down BTG1 expression. Transient transfection of BTG1 siRNA efficiently inhibited BTG1 expression in 786-O cells at 24, 48 and 72 h (Figure 3A). Cell viability assay showed that a treatment with BTG1 siRNA before irradiation promoted the decrease of cell viability induced by 5 Gy of X-rays (Figure 3B). Caspase-3 activity in cells treated with siRNA against BTG1 was increased by more than 3-fold as compared with cells treated with negative control (NC) group (Figure 3C). The survival fraction was also tested in 786-O cells after exposure to 5 Gy of X-rays. As expected, the colony formation efficiency of cells transfected with *BTG1* siRNA was significantly lower than that transfected with its negative control (Figure 3D and E). Furthermore, transfection with BTG1 siRNA led to a specific increase in the sub-G1 peak in 786-O cells at 48 h after IR treatment (Additional file 1D). These results suggest that down-regulation of *BTG1* significantly increases cellular radiation sensitivity in 786-O cells.

### BTG1 is a direct target of miR-454-3p

To analyze whether *BTG1* can be regulated by miRNAs, we used starBase (sRNA target Base), a miRNA-mRNA interaction prediction software consisting of five different algorithms, to predict the miRNAs which may potentially target the 3′-UTR of the *BTG1* mRNA [35]. We selected the miRNAs that were predicted to target *BTG1* by four or more algorithms for further analysis. These miRNAs included hsa-mir-130, hsa-mir-301a, hsa-mir-302, hsa-mir-454-3p, and hsa-mir-19b. Next, we constructed a series of pcDNA3.0 vectors that over-express these miRNAs and screened for those that regulate endogenous BTG1 levels at 48 h after transfection in 786-O cells. The *BTG1* protein levels in samples transfected with pcDNA3.0-miR-454-3p displayed a significant down-regulation (Additional file 2F), indicating that miR-454-3p can target *BTG1*. A reproducible repression of endogenous *BTG1* protein was also observed for vectors expressing miR-19b and miR-130, suggesting the possibility that multiple miRNAs target *BTG1*.

**Figure 4:**
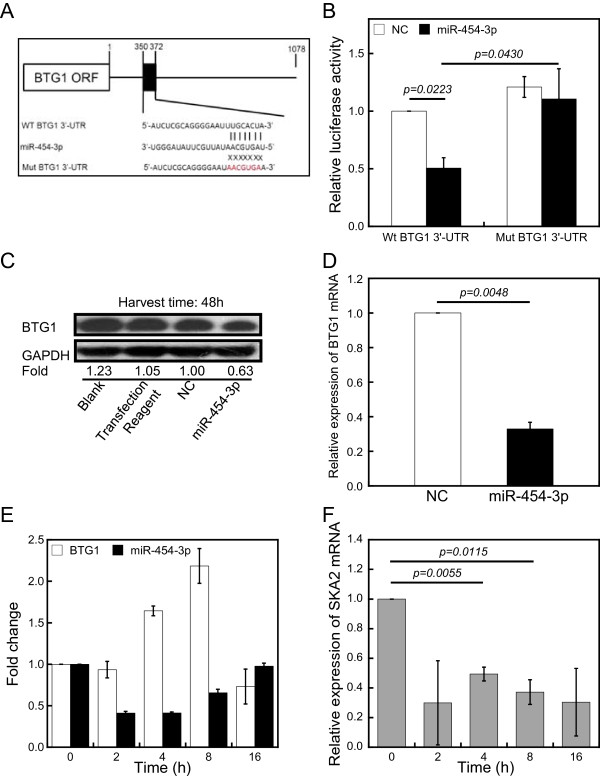
**MiR-454-3p negatively regulates BTG1 expression. (A)** Construction of a vector with either the wild-type sequence of the miR-454-3p binding site within the 3′-UTR of BTG1 (Wt BTG1 3′-UTR) or a mutated seed sequence (Mut BTG1 3′-UTR). The black box shows the predicted miR-454-3p binding site. The seed sequence is shown in red. **(B)** Luciferase reporter assay results are shown at 48 h following co-transfection of 786-O cells with the Wt BTG1 or Mut BTG1 vectors together with miR-454-3p mimic (miR-454-3p) or nonsense small RNA oligonucleotides (negative control, NC). Results (means ± SE) are representative for three independent experiments. **(C)** Western blotting was performed at 48 h after transfection with miR-454-3p or NC oligonucleotides. GAPDH was used as loading control. Fold changes of protein levels represent the average of three separate blots. **(D)** BTG1 expression is regulated by miR-454-3p at the mRNA level. QRT-PCR was conducted to quantify the expression level of BTG1 mRNA at 48 h after 786-O cells were transfected with miR-454-3p or NC oligonucleotides. **(E)** MiR-454-3p and BTG1 mRNA levels were monitored by qRT-PCR at the indicated time points after irradiation with 5 Gy of X-rays. U6 and GAPDH were used as controls. **(F)** *SKA2* mRNA levels were monitored by qRT-PCR at the indicated times after irradiation with 5 Gy of X-rays. GAPDH was used as a control. The data (means ± SE) are representative for at least four independent experiments with similar results.

To determine whether miR-454-3p directly targets *BTG1* via its 3′-UTR, the wild type and a mutant of BTG1 3′-UTR fragment with substitution in the seed region were synthesized and inserted into a luciferase report system (Figure 4A). The activity of the wild type reporter was significantly reduced by miR-454-3p mimic, whereas the miR-454-3p mimic had little effect on the mutant of BTG1 3′-UTR construct (Figure 4B). To further validate these findings, endogenous BTG1 levels were assessed in 786-O cells after transfection with miR-454-3p mimic. The miR-454-3p mimic specifically suppressed both the *BTG1* protein and mRNA levels at 48 h post-transfection (Figure 4C and D). These results confirm that *BTG1* is a target of miR-454-3p.

To determine whether endogenous miR-454-3p levels are regulated physiologically by IR, we assessed miR-454-3p expression in 786-O cells over a timecourse of exposure to 5 Gy of X-rays. While *BTG1* mRNA levels increased, miR-454-3p levels decreased upon treatment (Figure 4E). It is worth noting that the *BTG1* mRNA level decreased at 16 h after irradiation, whereas the miR-454-3p level increased.

The miR-454-3p gene is located in the first intron of the host gene, *SKA2* (*spindle and kinetochore-associated protein 2*), which plays a critical role in proper mitotic progression, checkpoint silencing, and timely anaphase onset [36]. To test whether the expression of *SKA2* correlates with that of miR-454-3p, *SKA2* mRNA was quantified by qRT-PCR. Similar to the results for miR-454-3p, the expression of *SKA2* was down-regulated in 786-O cells after IR (Figure 4F), providing further verification of our findings. These results indicate that miR-454-3p regulates the expression of *BTG1* through a direct interaction with the 3′-UTR of the BTG1, and that a decrease in its expression correlates with BTG1 up-regulation during IR treatment of renal carcinoma cells.

### Suppression of BTG1 by miR-454-3p enhances cell sensitivity to IR and relieves the G2/M arrest induced by IR in 786-O cells

**Figure 5:**
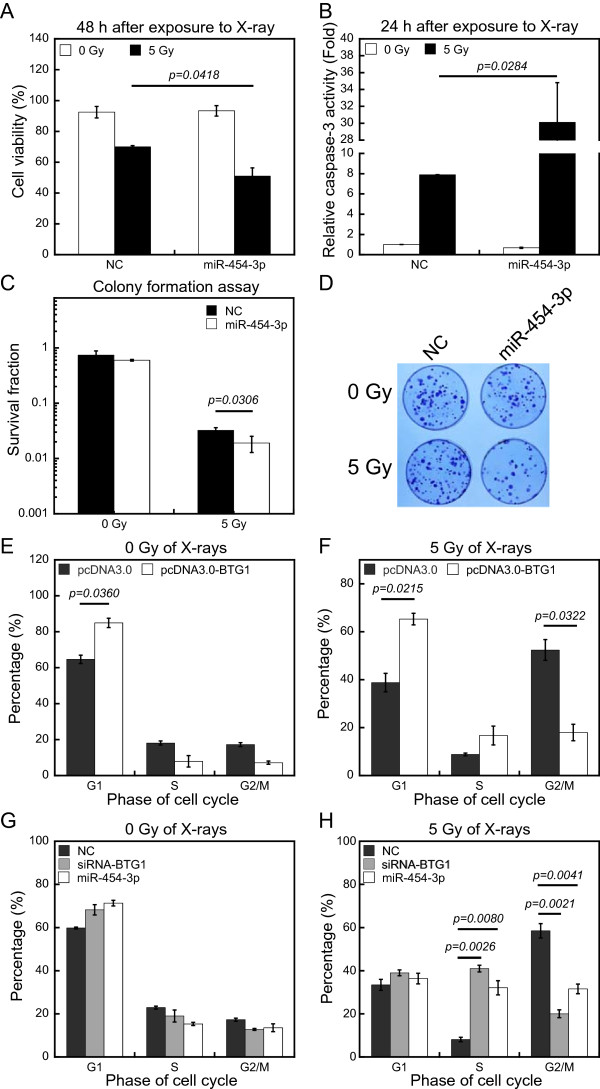
**MiR-454-3p enhances apoptosis and affects the G2/M arrest induced by IR. (A)** The percentage of cell viability of 786-O cells at 48 h after transfection of nonsense control siRNA (negative control, NC) or miR-454-3p mimic (miR-454-3p) was calculated by trypan blue dye exclusion assay with or without exposure to X-rays (48 h after exposure to X-ray). **(B)** The activation of caspase-3 were tested at 24 h in 786-O cells after transfection with nonsense control siRNA (negative control, NC) or miR-454-3p mimic (miR-454-3p) with or without exposure to X-ray (24 h after exposure to X-ray). **(C)** The effect of transfection with miR-454-3p mimic on clonogenicity of 786-O cells was characterized by colony formation assay. **(D)** Photographs of the colony formation of 786-O cells after various treatments. **(E and F)** 786-O cells were transfected with the pcDNA3.0 vector or pcDNA3.0-BTG1 vector for 48 h were analyzed for cell cycle kinetics **(E)** without irradiation (0 Gy of X-rays) or **(F)** with irradiation with 5 Gy of X-rays (5 Gy of X-rays). (G and H) 786-O cells transfected with control siRNA (NC), siRNA oligonucleotides against BTG1 (siRNA-BTG1), or miR-454-3p mimic (miR-454-3p) for 48 h were analyzed for cell cycle kinetics **(G)** without irradiation (0 Gy of X-rays) or **(H)** with irradiation with 5 Gy of X-rays (5 Gy of X-rays). The percentage of total cells at G0/G1, S, and G2/M phases were determined. The data (means ± SE) are representative for at least five independent experiments.

Our results thus far suggest that down-regulation of BTG1 can enhance the radiosensitivity of 786-O cells and that *BTG1* is also a target of miR-454-3p. Consequently, we postulated that miR-454-3p may affect cell radiosensitivity. To assess the effects of miR-454-3p on radiosensitivity, we exposed 786-O cells to 5 Gy of X-rays following transfection with miR-454-3p mimic. Trypan blue assay showed that the cell viability of miR-454-3p mimic transfected group relative to the NC group was reduced by 20% (Figure 5A). Similarly, over-expression of miR-454-3p mimic was associated with more than 4-fold increase of caspase-3 activity at 24 h (Figure 5B). Clonogenicity of 786-O cells transfected with miR-454-3p mimic was significantly reduced after exposure to 5 Gy of X-rays (Figure 5C and D). The sub-G1 percentage increased evidently at 48 h as assessed by flow cytometry (Additional file 1E), indicating that miR-454-3p enhances IR-mediated apoptosis.

Because cell cycle control is intimately involved in the induction of apoptosis during IR, we also examined whether BTG1 can regulate the cell cycle. 786-O cells were transfected with control pcDNA3.0 vector or pcDNA3.0-BTG1 expression vector, and the effects of IR on the cell cycle distribution were assessed by flow cytometry after 48 h. The majority of the cells were in the G1 phase prior to IR treatment. Exposure to 5 Gy of X-rays caused a strong G2/M arrest for cells transfected with control pcDNA3.0 vector (Figure 5E and F). Whereas, cells transfected with pcDNA3.0-BTG1 were resistant to IR-induced G2/M phase arrest after exposure to 5 Gy of X-rays. These results indicate that high levels of BTG1 can inhibit IR-induced G2/M arrest.

To further understand the role of BTG1 in inhibiting IR-induced G2/M arrest and to assess the effects of miR-454-3p on cell cycle arrest, 786-O cells were transfected with either siRNA oligonucleotides against BTG1 or miR-454-3p mimic. Flow cytometry analysis showed that BTG1 siRNA and miR-454-3p had no significant effect on cell cycle distribution in the absence of irradiation (Figure 5G). Surprisingly, however, BTG1 siRNA and miR-454-3p mimic promoted an IR-induced S phase arrest and abolished the G2/M arrest in 786-O cells exposed to 5 Gy of X-rays (Figure 5H). These results indicate that over-expression of BTG1 overcomes the G2/M arrest induced by IR, but knockdown of BTG1 expression causes a switch of the arrest from the G2/M phase to the S phase. One can easily conjecture about that down-regulation of BTG1 would impede the activation of the G1 checkpoint and lead to damage accumulation during S phase and the consequent triggering of the S phase arrest.

## Discussion

In the present study, we identified miR-454-3p as regulator of *BTG1* expression based on bioinfomatic software prediction and detailed experimental validation. The expression of *BTG1* and miR-454-3p was shown to inversely correlate in 786-O cells upon exposure to environmental stressors, thus supporting a physiological role for miR-454-3p in regulating BTG1 expression. In addition to miR-454-3p, miR-19b was predicted to have two binding sites on the 3′-UTR of the BTG1 mRNA, one of which occupies the same site as the putative miR-454-3p target. A significant repression of endogenous BTG1 protein by miR-19b expression vector was also observed in 786-O cells (Additional file 2F). Therefore, we speculate that more than one miRNA may target BTG1, and these miRNAs may function antagonistically under specific physiological conditions. Future experimentation to address this possibility may unveil a regulation network of BTG1 regulation by miRNAs.

Our results show that the expression of *SKA2* is regulated coordinately with the expression of miR-454-3p. Interestingly, miR-454-3p and a third putative BTG1 target, miR-301a, are encoded within the first intron of *SKA2*, whose depletion affects the cell cycle by inducing a metaphase-like delay [37]. MiR-301a also down-regulates the expression of an inhibitor of NF-κB, Nkrf [38]. NF-κB binds directly to the *SKA2* promoter region to activate the transcription of *SKA2* and miR-301a and also enhances persistent NF-κB activation to facilitate tumor growth [37, 39], thus suggesting a feedback loop to moderate SKA2 function. It is intriguing that miR-454-3p targets BTG1, which has a strong anti-proliferative ability, and suggesting an additional complexity of the growth regulatory function derived from the *SKA2* locus. BTG1 has been suggested to inhibit NF-κB activities [40]. Therefore, complex regulatory loop appears to regulate cell growth inhibition by BTG1, and it is likely that SKA2, NF-κB and multiple miRNAs are coordinated to control the BTG1 expression.

Little is known about the molecular mechanisms of control of the cell cycle by BTG1, and most of the information comes from interaction studies. BTG1 exerts cellular functions by interacting with PRMT1, HOXB9, and hCAF1, which regulate the expression of a number of genes involved in cell cycle control and progression [41–43]. CCNA2 (cyclinA2) physically associates with BTG1 [44] and controls S phase by activating CDK2 kinases to initiate DNA synthesis [45]. Ectopic over-expression of CCNA2 triggers checkpoint response and subsequently increases the S phase population in mammalian cells [45]. We found that siRNA-mediated silencing of BTG1 led to S phase arrest after IR. Additionally, the S phase arrest can also be mediated by miR-454-3p. Furthermore, miR-454-3p is highly expressed in the S phase. The above negative regulation may suggest the association between CCNA2 and BTG1 in the control of the cell cycle progression of S phase. However, our results also show that over-expression of BTG1 in 786-O cells promotes a G1 arrest. Therefore, the link between miR-454-3p, BTG1 and cell cycle is likely complex and warrants further investigation to unravel the network of cell cycle regulators that are functionally associated with BTG1.

## Conclusions

In conclusion, our study reveals that *BTG1* is a direct target of miR-454-3p. Changes of its expression levels render tumor cells sensitive to radiation due to the role of BTG1 in cell cycle progression. Therefore, down-regulation of BTG1 via miR-454-3p presents a possible strategy to sensitize tumor cells to radiotherapy.

## Electronic supplementary material

Additional file 1: **(A)** **The graph represents the caspase-3 colorimetric calibration, which were measured with an ELISA reader at an absorbance of 405 nm (Caspase-3 colorimetric calibration).** Results are representative for three independent experiments. **(B)** The dynamic of caspase-3 activity was tested in 786-O cells without transfection after exposure to 5 Gy of X-rays (Activation of caspase-3 by X-ray). Results are representative for three independent experiments. (**C**, **D** and **E**) The sub-G1 percentage of all the treatments of 786-O cells were analyzed by flow cytometry 48 h after X-ray exposure. Results are representative for five independent experiments. (PPTX 111 KB)

Additional file 2: **(F) Western blot showing the protein levels of BTG1 in 786-O cells 48 h after transfection with pcDNA3.0 miRNA vectors.** GAPDH is shown as loading control. Fold changes of protein levels represent the average of three separate blots. (PPTX 140 KB)
