# Supplementary figures and images for: Down-regulation of BTG1 by miR-454-3p enhances cellular radiosensitivity in renal carcinoma cells
Source: Radiat Oncol. 2014 Aug 12;9:179. doi: 10.1186/1748-717X-9-179 (PMC4252025; doi:10.1186/1748-717X-9-179)

## Slide 1
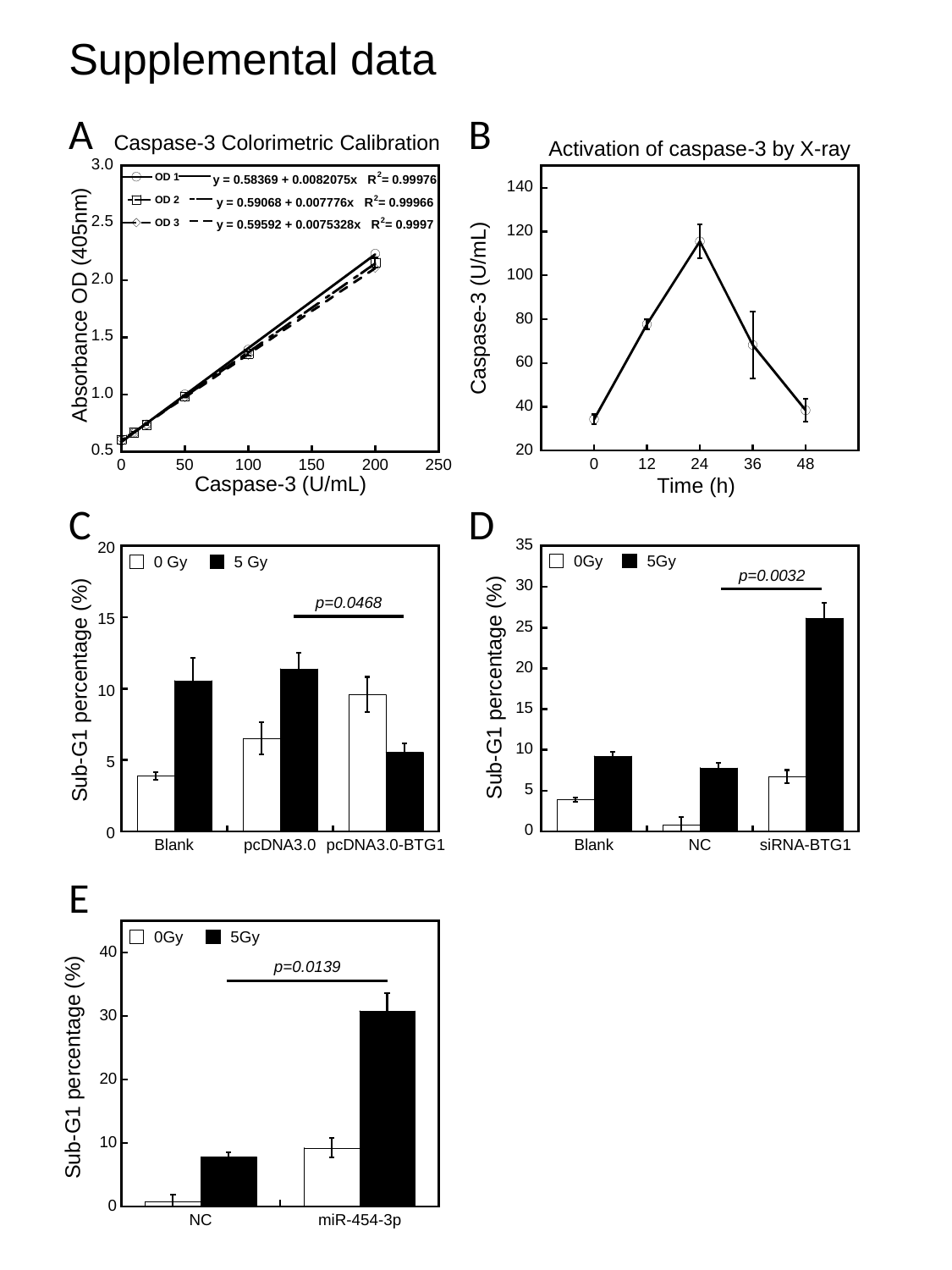

Supplemental data
A
B
C
D
E

Supplement: Supplementary file 1 — Additional file 1: (A) The graph represents the caspase-3 colorimetric calibration, which were measured with an ELISA reader at an absorbance of 405 nm (Caspase-3 colorimetric calibration). Results are representative for three independent experiments. (B) The dynamic of caspase-3 activity was tested in 786-O cells without transfection after exposure to 5 Gy of X-rays (Activation of caspase-3 by X-ray). Results are representative for three independent experiments. (C, D and E) The sub-G1 percentage of all the treatments of 786-O cells were analyzed by flow cytometry 48 h after X-ray exposure. Results are representative for five independent experiments. (PPTX 111 KB) [file 13014_2014_1146_MOESM1_ESM.pptx]
